# The Effects of Different Molding Orientations, Highly Accelerated Aging, and Water Absorption on the Flexural Strength of Polyether Ether Ketone (PEEK) Fabricated by Fused Deposition Modeling

**DOI:** 10.3390/polym15071602

**Published:** 2023-03-23

**Authors:** Daisuke Miura, Yoshiki Ishida, Akikazu Shinya

**Affiliations:** 1 Department of Dental Materials Science, School of Life Dentistry at Tokyo, The Nippon Dental University, 1-9-20 Fujimi, Chiyoda-ku, Tokyo 102-8159, Japan; 2 Turku Biomaterials Research Program, Department of Biomaterials Science, Institute of Dentistry and BioCity, University of Turku, 20500 Turku, Finland

**Keywords:** polyether ether ketone, highly accelerated aging, fused deposition modeling, bending strength, water absorption, molding orientations

## Abstract

Rising prices are currently a problem in the world. In particular, the abnormal increases in the price of metals, which are often used in dental prosthetics, have increased the burden of dental costs on the public. There is therefore an urgent need to develop prosthetic devices made from materials that are not affected by the global situation and that have excellent biocompatibility and mechanical properties comparable to those of metals. Polyether ether ketone (PEEK) is a promising alternative to metal in dentistry. This study compared the effects of different molding orientations, highly accelerated aging, and water absorption on the flexural strength of PEEK fabricated by fused deposition modeling (FDM) and examined its potential for dental applications. The flexural strength of PEEK stacked at 0° to the molding stage (0° PF), with and without highly accelerated aging, was significantly greater than for the other molding orientations. As with PD, the maximum test load for 0° PF was measured without fracture. PEEK stacked at 45° (45° PF) and 90° (90° PF) to the molding stage easily fractured, as the applied load pulled the stacked layers. No statistically significant difference was found between the flexural strength of 45° PF and 90° PF. The flexural strength decreased under all conditions due to defects in the crystal structure of PEEK caused by highly accelerated aging.

## 1. Introduction

In recent years, polyether ether ketone (PEEK) has been applied to the field of dentistry in Japan and abroad. PEEK is a super engineering plastic developed by Imperial Chemical Industries in England in 1977. Compared with commodity plastics, super engineering plastics are substitutes for metal materials in general environments. They have superior heat resistance above 100 °C and good resistance to impacts, creep, and chemicals. Plastics that can withstand temperatures of 150 °C or higher are called super engineering plastics, and PEEK has high long-term heat resistance at 250 °C [1]. Due to its excellent heat resistance and stability during sterilization, PEEK is used as a base material for surgical instruments and carbon-fiber-reinforced composites [2,3,4,5,6,7,8], and it is applied in artificial joints, bone replacement, bone fixation, and dental implants. Its effectiveness has been recognized in some cases, and it is being used clinically. PEEK has high fatigue resistance and toughness, and it does not elute ions, which is a problem with metallic materials. Therefore, PEEK is being considered for dental prosthetic applications, such as fixtures and abutments for dental implants, orthodontic wires, denture clasps, crowns, and bridges. In dentistry, computer-aided design (CAD)/computer-aided manufacturing (CAM) is mainly used to cut PEEK using computer-controlled machining processes and rotary cutting tools, such as ball end mills and diamond points, based on input data. The shape of the material to be machined can be a block or a disk. The quality of CAD/CAM modeling is not dependent on the skill level of the maker, and therefore, the quality can be kept constant. Dental CAD/CAM systems consist of a personal computer and milling machine, together with a scanner and monitor. The scanner head is positioned intraorally over the tooth preparation, and the resulting data are displayed on the monitor as a two-dimensional (2D) or three-dimensional (3D) image. The design work is carried out on the monitor, and instructions are sent to a computer-controlled milling machine. The restoration is milled from a block or disc. Materials that can be used include feldspar, leucite, lithium dislocates, and composite blocks. However, although PEEK has the highest performance among the thermoplastic resins, with excellent resistance to heat, chemicals, hot water, and flame, and excellent mechanical and electrical properties, it is extremely expensive (approximately 10,000 JPY/kg), and cutting by CAD/CAM wastes a large amount of material. In addition, the size and shape of molds that can be fabricated are limited [9,10,11,12,13,14,15,16,17,18,19,20,21]. A 3D printer is a device that, given shape data about an object, forms the object based on those data. The object is formed by layering the cross-sectional shape of the object. The principle is fundamentally different from the slicing and modeling method, in which the desired object is obtained by cutting the material. Additive manufacturing (AM) is a molding method in which layers of material are stacked to form an object, generally using a 3D printer. The technology is based on depositing 2D sliced layers on top of each other to create 3D objects. This fabrication method produces layers with cross-sections defined by data and then stacks them. This method is efficient in terms of energy and material usage and is not limited by tool size or cutting angle. Therefore, it is possible to precisely fabricate complex forms in a way that was not possible in the past [22,23]. There are seven molding methods applicable to dentistry: sheet lamination, vat photopolymerization, material jetting, binder jetting, powder bed fusion, directed energy deposition, and material extrusion. Sheet laminating is a forming process in which thin sheets of materials such as paper, resin, and metal are cut into layers according to contours that match the cross-sectional shape of the layer, which are repeatedly joined and laminated until the designed shape is achieved. Vat photopolymerization is a modeling method in which the liquid light-curing resin stored in a tank is irradiated with a laser or other means to cure the necessary areas and then layered. Material jetting is a modeling technique that allows complex objects to be modeled by injecting model and support materials from a nozzle onto a stage, which is then cured by UV radiation, heating, or cooling, depending on the material. The binder jetting solidifies metal powders by injecting a liquid binder through a nozzle. Powder bed fusion is a process in which thermal energy is used to selectively melt and bond specific areas of a powder bed. Directed energy deposition uses concentrated thermal energy at a single point to melt and fuse metallic materials where they are placed. In the thermal melting and laminating (FDM) method, a wire filament of polylactic acid resin (PLA) or acrylonitrile butadiene styrene (ABS) resin with a diameter of approximately 2 to 3 mm is thermally melted and extruded through a narrow nozzle. The stage is then lowered by a certain amount, and the thermally melted resin is extruded again. This method is used to produce models and cast patterns because the equipment is relatively inexpensive. FDM is also considered the most advanced and promising technology in AM [18,24]. However, in general, 3D printers have extrusion nozzle temperatures from approximately 200 °C to 270 °C, making it difficult to use heat-resistant PEEK for FDM [25,26]. Recently, there have been remarkable developments in 3D printers, and desktop-type 3D printers with nozzle temperature (450 °C) and ambient temperature (165 °C) required for the FDM of PEEK have appeared on the market. However, the desired mechanical properties of PEEK moldings fabricated by FDM for dental applications have not been fully investigated. In addition, FDM-fabricated moldings are stacks of layers with steps between layers. The anisotropy of the steps in the molding results in different mechanical properties. Therefore, researchers have studied the effects of this anisotropy on formed products and their mechanical properties [27,28,29,30,31]. However, there have been few studies on the use of molded products produced by FDM in the oral cavity. In this study, we compared the effects of different stacking directions, highly accelerated aging, and water absorption on the flexural strength of FDM PEEK (FMP) and investigated the effectiveness of FMP for dental applications.

## 2. Materials and Methods

The materials used in this study, PEEK for FDM (PEEK filament, INTAMSYS, Shanghai, China, PF) and discs of PEEK for CAD/CAM (PEEK Biosolution, Merz Pharma GmbH & Co, Frankfurt, Germany, PD) are shown in Table 1. The composition of PF was only polyether ether ketone, whereas PD, which was used as the control, already contained TiO_2_ as a component. The FDM 3D printer was a FUNMAT HT (INTAMSYS, Shanghai, China). The specimens were produced using the printing parameters specified by the manufacturer in Table 2.

### 2.1. Experiment 1: 3-Point Bending Test

**Figure 1:**
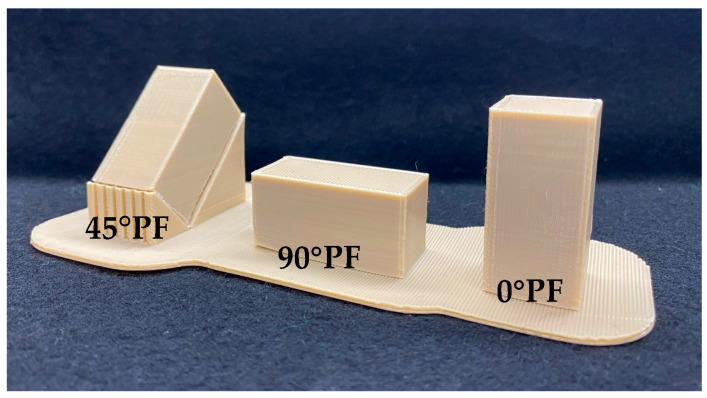
FDM-modeled PEEK (0° PF, 45° PF, and 90° PF).

Rectangle-shaped specimens (200 × 200 × 250 mm) were created using 3D modeling software (3D Builder, Microsoft, Redmond, WA, USA) and placed at angles of 0°, 45°, and 90° to the fabrication stage (0° PF, 45° PF, and 90° PF, respectively) by using 3D slicing software (INTAM-SUITE, INTAMSYS, Shanghai, China). Then, a rectangular sample was fabricated using a 3D printer and annealed at the temperature specified by the manufacturer using an air-cooled constant-temperature incubator (DKN602, Yamato Chemical Co., Tokyo, Japan), as shown in Figure 1. The annealing process was carried out according to a programed provided by the manufacturer; the FDM-modeled PEEK was placed on a stainless steel plate. It was first heated from room temperature to 90 °C for 2 h. It was then heated at 90 °C for four hours and then increased to 120 °C for one hour; heated at 120 °C for three hours and then increased to 150 °C for one hour; heated at 150 °C for eight hours and then increased to 200 °C for two hours; heated at 200 °C for six hours and then decreased to 150 °C for four hours; heated at 200 °C for four hours and then increased to 150 °C for one hour; heated at 150 °C for eight hours and then increased to 150 °C for two hours; heated at 200 °C for six hours and then decreased to 200 °C for four hours; heated at 150 °C for four hours and then increased to 150 °C for two hours; heated at 150 °C for 4 h and returned to room temperature at the end of the process. The schedule for annealing as specified by the manufacturer is shown in Table 3. The bar specimens for the 3-point bending test were fabricated according to ISO-10477. The fabricated rectangular PEEK specimens were cut into bars 2 mm wide × 2 mm thick × 25 mm long on a precision cutting machine (Isomet 1000, Buehler, Lake Bluff, IL, USA). The bars were then polished using 1 µm alumina and stored in deionized water at 37 ± 2 °C for 50 ± 2 h. For highly accelerated aging, the rod specimens were placed in a test apparatus (PC-242HS-A, Hirayama, Saitama, Japan) for 5 h at 100% humidity, 132 °C, and 1.2 atmosphere. Before and after highly accelerated aging, 3-point bending tests were performed on specimens using a universal testing machine (AGS-X, Shimadzu, Kyoto, Japan) (crosshead speed 1 mm/min, radius of curvature of load-bearing part 1.0 mm, support roller diameter 2 mm, and distance between fulcrums 20 mm). The flexural strength σ (MPa) is represented by Equation (1), where P (N) is the load at failure; L (mm) is the center-to-center distance between the supporting rollers; and w (mm) and t (mm) are the width and thickness of the specimen, respectively.

σ = 3 PL/2 wt^2^ (1)

The number of repetitions for each 3-point bending test was set to 10 (*n* = 10), and one-way analysis of variance (ANOVA) and Tukey’s pairwise comparisons were performed for each of the flexural strength and flexural modulus values obtained.

### 2.2. Experiment 2: Water Absorption and Dissolution Test

Cylinders-shaped specimens (diameter 150 × height 200 mm) were created using 3D modeling software and placed at angles of 0°, 45°, and 90° to the fabrication stage (0° PF, 45° PF, and 90° PF, respectively) by using 3D slicing software. The cylindrical specimens were fabricated using a 3D printer and annealed at the temperature specified by the manufacturer using an air-cooled constant-temperature incubator. For the water absorption test, disk samples were cut from a cylinder according to ISO-10477. The fabricated cylindrical PEEK samples were cut into disks 1.5 mm in diameter × 1 mm in thickness using a precision cutting machine. The fabricated cylindrical PEEK specimens were then polished using 1 µm alumina. The specimens were then stored in deionized water at 37 ± 1 °C. After 22 h, the specimens were removed and stored in a desiccator at 23 ± 2 °C for 2 h. Each specimen was then weighed. Microbalances (Sartorius MC 210 S, Sartorius, Göttingen, Germany) were used for the measurements. During measurement, the object to be measured was not held directly in the hand but was moved in and out using long tweezers. In order to eliminate errors, the measured object was placed in the weighing chamber of the balance for several hours before the measurement, and only when the temperature of the measured object had reached the same temperature (20 °C) as that in the balance was it placed on the weighing pan and measured. And the final mass m1 was determined when the specimen’s mass loss was less than 0.1 mg within 24 h. The specimens were then stored in 20 mL of deionized water at 37 ± 1 °C for 7 days, and the mass removed was determined as m2. After weighing, the final mass of the specimen, which was dried again, was m3. The water absorption and dissolution of each specimen were determined by the following Equations (2) and (3).

Pws (water absorption, µg/mm^3^) = m^2^ (mass of specimen after 7 days in water, µg)—m^3^ (mass of specimen dried again after storage in water, µg)/V (volume of specimen, mm^3^) (2)

Psl (dissolved volume, µg/mm^3^) = m^1^ (mass of impressed specimen before storage in water, µg)—m^3^ (mass of dried specimen after storage in water, µg)/V (volume of specimen, mm^3^) (3)

One-way ANOVA and Tukey’s pairwise comparisons were performed for each of the obtained water absorption and dissolution values.

### 2.3. Experiment 3: Scanning Electron Microscopy (SEM)

The microstructures were observed using SEM (JSM-IT200, JEOL, Tokyo, Japan) with an accelerating voltage of 15.0 kV. Three different molding orientations (0° PF, 45° PF, and 90° PF) were observed at 200×. The results showed no significant differences in the lamination planes.

All experiments were performed by the same person.

## 3. Results

**Figure 2:**
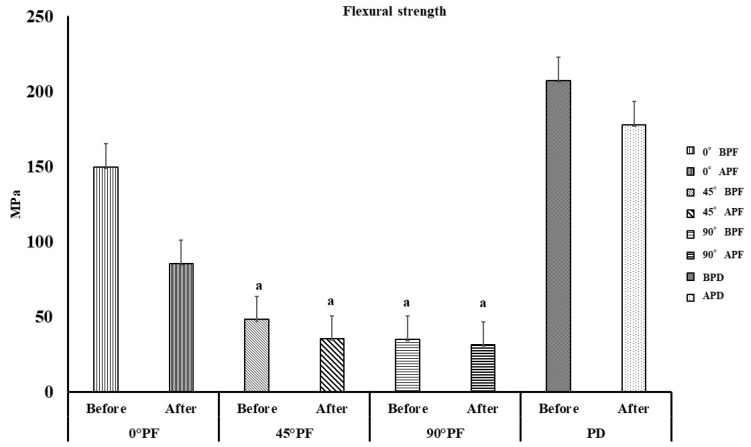
Flexural strength in 3-point bending tests (*p* < 0.05, I: 95% confidence interval standard deviation); there is no statistically significant difference between the results indicated by “a”.

The results of the three-point bending test are shown in Figure 2. The PF and PD before and after highly accelerated aging are denoted BPF and BPD and APF and APD, respectively. The flexural strength was in the following order: BPD > APD > 0° BPF > 0° APF > 45° BPF > 45° APF ≧ 90°BPF ≧ 90° APF. BPD exhibited the highest flexural strength of 207.2 MPa, while 90°AFA exhibited the lowest value of 31.2 MPa. Additionally, 0° PF showed a statistically significant difference (*p* < 0.05) in flexural strength before and after the highly accelerated aging. This was the same behavior as the control, PD. No difference in flexural strength was observed before and after highly accelerated aging for 45° PF and 90° PF (*p* > 0.05). The comparison between the molding orientations before highly accelerated aging showed that 0° BPF (145 MPa) had the largest value. This was followed by 45° BPF (48.0 MPa) and 90° BPF (34.9 MPa). Statistically significant differences in the 0° BPF values were found among all samples (*p* < 0.05), and no differences were found between 45° BPF and 90° BPF (*p* > 0.05). The maximum test load value was measured at 0° BPF without breaking. This behavior was similar to that of BPD. Comparison of the molding orientations after the highly accelerated aging showed that 0° APF (85.4 MPa) had the highest value, as was the case before highly accelerated aging. This was followed by 45° APF (35.2 MPa) and 90° APF (31.2). The maximum test load value was measured without 0° APF rupture. This was similar to the 0° BPF results.

**Figure 3:**
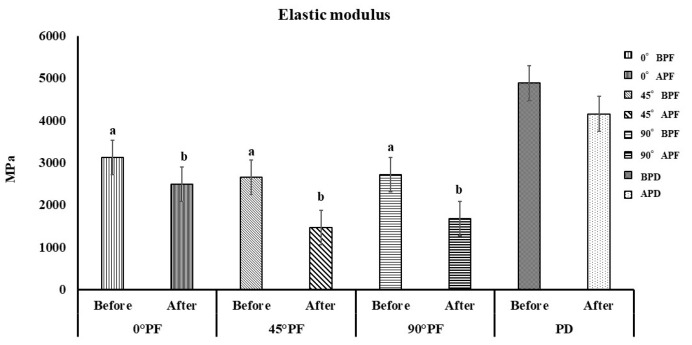
Elastic modulus in 3-point bending tests (*p* < 0.05, I: 95% confidence interval standard deviation); there is no statistically significant difference between results indicated by “a” or by “b”.

The results for the elastic modulus are shown in Figure 3. The order of elastic modulus was BPD > APD > 0° BPF > 90° BPF > 45° BPF > 0 APF > 90° APF > 45° APF. The elastic modulus of BPD was the largest at 4884 MPa, while that of 90°AFA was the smallest at 1466 MPa. There was a statistically significant difference in the elastic modulus before and after the highly accelerated aging for all different molding orientations (*p* < 0.05). However, a comparison of the molding orientations before highly accelerated aging showed no significant differences among all conditions except PD, which was almost constant regardless of the molding orientation (*p* > 0.05).

**Figure 4:**
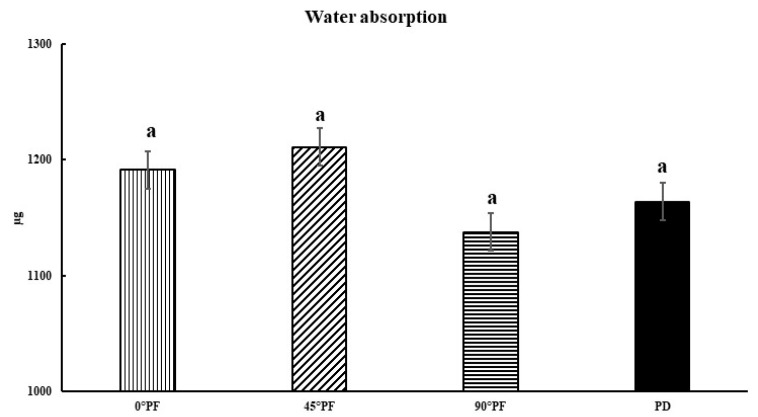
Water absorption results (*p* < 0.05, I: 95% confidence interval standard deviation); there is no statistically significant difference between results indicated by “a”.

**Figure 5:**
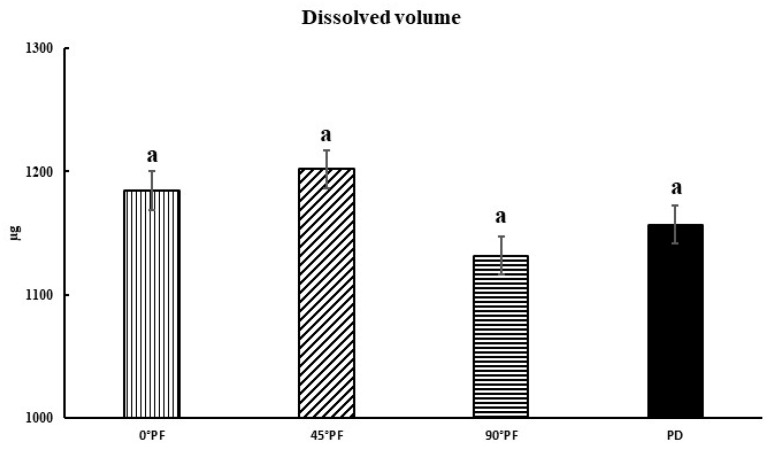
Dissolution volume results (*p* < 0.05, I: 95% confidence interval standard deviation); there is no statistically significant difference between results indicated by “a”.

The comparisons of the molding orientations after highly accelerated aging also showed no significant differences in values between all conditions except PD (*p* > 0.05). The stresses generated by the bending load were compressive at the top of the specimen and tensile at the bottom; 0° PF was considered to have not cracked as a result of the forces perpendicular to the stacking direction, with or without high accelerated aging treatment. The results for water absorption and dissolution are shown in Figure 4 and Figure 5. Water absorption was in the order 45° PF > 0° PF > PD > 90° PF, with 45° PF having the largest value (1211 μg) and 90° PF having the smallest value (1137 μg). There was no significant difference in water absorption in any molding orientation. The order of dissolution was 45° PF > 0° PF > PD > 90° PF, with 45° PF having the largest value (1202 μg). There were no significant differences in dissolution among the molding orientations. These results suggest that water absorption and dissolution volume are independent of the stacking direction.

## 4. Discussion

**Figure 6:**
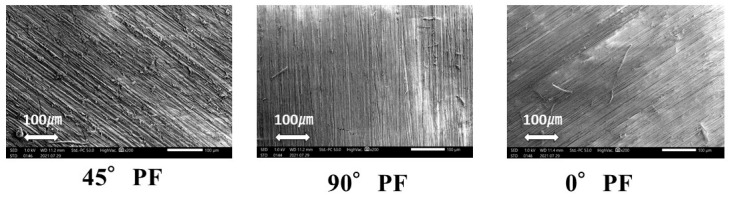
SEM images of FDM of PEEK.

PEEK has been considered for application to various prosthetic devices because of its flexural strength, corrosion resistance, and biocompatibility. However, the current mainstream method of PEEK molding in dentistry is the machining method. Cutting removes expensive PEEK, which is a waste of material. However, the greatest advantage of the AM method is that it does not require a model for molding. In addition, it can be directly fabricated from digital 3D data. Compared with the machining method, this method is suitable for modeling PEEK because there is less material waste. In this study, the effects of different molding orientations, highly accelerated aging, and water absorption on the mechanical properties of FDM-modeled PEEK were investigated. Electron microscopy images of the stacking planes of the three types of PFMs (0° PF, 45° PF, and 90° PF) are shown in Figure 6. Uniform PEEK molding surfaces were observed under all conditions. Therefore, there was no significant difference in the molding orientations.

As shown in Figure 1, the flexural strength decreased as the stacking angle increased with respect to the molding stage. Comparing the molding orientations before highly accelerated aging, 45° PF and 90° PF exhibited approximately 1/3 of the flexural strength of 0° PF. The 0° PF and control PD specimens reached the maximum load without breaking, with or without highly accelerated aging. However, the 45° PF and 90° PF specimens failed with or without highly accelerated aging. These results indicate that the bending behavior was the same for the 0° PF and PD specimens. These results may be attributed to the way the load was applied to the stacked surfaces due to the difference in the molding orientations. The 45° PF and 90° PF specimens easily failed due to the applied shear forces, which pulled away the steps causing the molding orientations. However, in the case of 0° PF, the force was applied perpendicular to the molding orientation, and the stress was distributed over the entire specimen, which resulted in a strong resistance to fracture, as in the case of PD. The flexural strength of FDM-modeled PEEK also involves the porosity, stiffness, anisotropy, and interlaminar bonding of the material. All of these are then considered to combine to influence the final flexural strength and fracture behavior. It is well known that the tensile stresses induced by bending play an important role in the failure of materials. In addition, the distribution of pores in the material is important for tensile stresses. Considering that the pores are distributed on the stacking planes of the specimens, 0° PF had a large flexural strength due to the distribution of pores along the stress direction. However, in 45° PF and 90° PF, the pores were distributed perpendicular to the stress, which we considered to have significantly reduced the specimen bearing capacity. In the future, the distribution of pores in different stacking directions should also be compared. Figure 2 shows that the elastic modulus was almost constant regardless of the molding orientations. However, highly accelerated aging reduced the elastic modulus by approximately 80% for 0° PF and by approximately 50% for 45° PF and 90° PF. This indicated that the FDM-modeled PEEK exhibited tougher behavior for smaller angles and more brittle behavior for larger angles in the molding orientations. The flexural strength and elastic modulus decreased in all molding orientations before and after highly accelerated aging. No significant differences in water absorption or dissolution were observed for the molding orientations. It was unlikely that moisture during highly accelerated aging had any effect on the physical properties of the FDM-modeled PEEK. This is because PEEK is a crystalline resin; during annealing, it is heated above its glass transition temperature. The glass transition temperature of PEEK is 143 °C, which is higher than the boiling point of water. The expansion of water in PEEK causes problems with crystallization [32,33,34]. Highly accelerated aging was performed at 100% humidity, 132 °C, and 1.2 atmosphere. It caused defects in the crystal structure of the FDM-modeled PEEK, which resulted in a decrease in flexural strength under all conditions. Li et al. performed bending tests on FDM carbon-reinforced PEEK composites [35]. They found that specimens molding horizontally to the stage initially deformed both linearly and elastically and then reached maximum flexural stress without failure. Carbon-reinforced PEEK composite molding perpendicular to the stage suffered delamination failure and had 1/3 of the flexural strength of those molding horizontally. These results are also in line with the results of this study. Therefore, when using FDM of PEEK to fabricate prosthetics, such as bridges, that are deformed by intraoral occlusal forces, the molding orientations must be fully taken into account. However, the results for the elastic modulus before highly accelerated aging showed no difference with respect to the molding orientation; the elastic modulus was almost constant. This indicates that the same treatment can be applied to the fabrication of prosthetics with a small amount of deformation as with isotropic materials. There were some limitations in the experiment. PEEK can also be formed by powder bed fusion (PBF). In a study by Uehara et al., PEEK was produced by PBF and the mechanical properties of the product were evaluated [36]. PBF is a process for creating three-dimensional structures by depositing a thin layer of powder, selectively melting and solidifying it with a laser, and repeating the process. The flexural strength of PBF molding PEEK parts (90–95% fill content) was approximately 139 MPa. The flexural strength of the 0° PF before highly accelerated aging in this study was approximately 150 MPa, which was higher than that of the PBF molding PEEK. In PBF, the bottom surface is usually preheated to near melting point to prevent distortion of the molding. When molding with highly heat-resistant resins in PBF, high heat resistance is required for the molding equipment. Sparks and smoke are generated when modeling PEEK in PBF, and even at the same laser energy density, the obtained modeling density varies because the conditions for heating the powder bed differ if the scanning speed, spacing, shape of the modeled object, and scanning direction are different. Therefore, PEEK parts made by FDM are easy and stable to mold, and it is thought that future dental applications are also feasible. This study suggests that FDM-modeled PEEK has physical properties that are appropriate for dental materials. However, compatibility with abutment teeth and dimensional changes due to heat treatment, which are necessary for the clinical application of FDM-modeled PEEK, are still unclear. The PEEK produced by FDM accumulates residual stress inside. Because PEEK is a crystalline resin, the temperature of this heat treatment must be above the glass transition temperature. In this process, a large amount of shrinkage occurs. Therefore, it is essential to design moldings that take into account the shrinkage caused by heat treatment. Future research should consider the influence molding orientations on compatibility between prosthetics fabricated with FDM PEEK and abutment teeth and the design of prosthetic devices that take into account shrinkage of PEEK due to heat treatment.

## 5. Conclusions

In this study, PEEK was molded by FDM and various mechanical properties were compared and investigated with the following results.

(1) The maximum flexural strength of FDM PEEK objects fabricated at 0° to the molding stage was measured without fracture. The same was also true after highly accelerated aging. Apparently, when the force was applied perpendicular to the molding orientations, the stress was distributed over the entire specimen, which resulted in strong resistance to fracture.
(2) Highly accelerated aging degraded the mechanical properties of FDM PEEK objects due to defects in their crystal structure. This occurred in all stacking directions.
(3) No significant differences in water absorption or solubility were observed among the fabrication methods. Therefore, it was unlikely that moisture during the highly accelerated aging process affected the physical properties of FDM PEEK.

These results indicated that it is necessary to give sufficient consideration to the stacking direction when fabricating FDM PEEK prosthetics for applications where they will be deformed by intraoral occlusal forces.

## Figures and Tables

**Table 1:** Composite resins used in this study.

Code	Product Name	Component	LOT Number	Manufacture
PF	PEEK filament	Poly-ether-ether-ketone	202,653	INTAMSYS, Shanghai, China
PD	PEEK Biosolution	Poly-ether-ether-ketone TiO_2_	40,318	Merz Pharma GmbH & Co, Frankfurt, Germany

**Table 2:** Printing parameters.

Molding Conditions	
Stacking Pitch	0.1 mm
Nozzle system	0.4 mm
Infill	100%
Infill pattern	line
Material	PEEK
Speed	60 mm/s
Fan speed	50%
Support overhang angle	50°
Support distance	0.1 mm
Build plate adhesion type	Raft
Nozzle temperature	410 °C
Chamber temperature	90 °C

**Table 3:** Annealing process schedule.

	Temperature (°C)	Setting Time (h)
Step 1	90	2
Step 2	90	4
Step 3	120	1
Step 4	120	3
Step 5	150	1
Step 6	150	8
Step 7	200	2
Step 8	200	6
Step 9	150	4
Step 10	150	4
Step 11	0	0.5

## Data Availability

The data presented in this study are available through email upon request to the corresponding author.
